# Nonfatal Violent Workplace Crime Characteristics and Rates by Occupation — United States, 2007–2015

**DOI:** 10.15585/mmwr.mm6912a2

**Published:** 2020-03-27

**Authors:** Miriam Siegel, Candice Y. Johnson, Christina C. Lawson, Marilyn Ridenour, Daniel Hartley

**Affiliations:** ^1^Division of Field Studies and Engineering, National Institute for Occupational Safety and Health, CDC; ^2^Epidemic Intelligence Service, CDC; ^3^Division of Safety Research, National Institute for Occupational Safety and Health, CDC.

Workplace violence can lead to adverse physical and psychological outcomes and affect work function (*1*). According to the U.S. Bureau of Labor Statistics, intentional injury by another person is a leading cause of nonfatal injury requiring missed workdays (*2*). Most estimates of workplace violence include only crimes reported to employers or police, which are known underestimates (*3*,*4*). Using 2007–2015 data from the National Crime Victimization Survey (NCVS), characteristics of self-reported nonfatal violent workplace crimes, whether reported to authorities or not, and rates by occupation were examined. Estimates of crime prevalence were stratified by crime characteristics and 22 occupational groups. Overall, approximately eight violent workplace crimes were reported per 1,000 workers. During 2007–2010, workers in Protective services reported the highest rates of violent workplace crimes (101 per 1,000 workers), followed by Community and social services (19 per 1,000). Rates were higher among men (nine per 1,000) than among women (six per 1,000). Fifty-eight percent of crimes were not reported to police. More crimes against women than against men involved offenders known from the workplace (34% versus 19%). High-risk occupations appear to be those involving interpersonal contact with persons who might be violent, upset, or vulnerable. Training and controls should emphasize how employers and employees can recognize and manage specific risk factors in prevention programs. In addition, workplace violence-reduction interventions might benefit from curricula developed for men and women in specific occupational groups.

Data were analyzed from the Bureau of Justice Statistics’ NCVS, a national survey of self-reported victimizations in the United States (*5*).* The U.S. Census Bureau administers NCVS to collect information on nonfatal crimes through in-person or telephone interviews of persons aged ≥12 years from a nationally representative household sample. A sample is identified through a stratified, multistage sampling design; annual response rates typically range from 80% to 90% (*6*). Respondents are asked to report crimes they experienced during the preceding 6 months. The years 2007–2015 represent the most recently available period for which data were collected with comparable sampling strategies.

Incidents included in the analysis were self-reported to have occurred while victims, aged ≥16 years, were working or on duty in the United States. Types of crime analyzed included five mutually exclusive categories: rape/sexual assault, robbery, aggravated assault, simple assault, and verbal threat of assault.^†^ Free-text survey responses on occupation at the time of the workplace crime were categorized by NCVS into 44 nonmilitary occupational groups. These occupations were collapsed into 22 major groups defined by the U.S. Census Bureau.^§^

To describe violent workplace crimes, weighted prevalence estimates stratified by crime characteristics were calculated along with 95% confidence intervals (CIs) estimated using Taylor series linearization. Estimates were stratified by victim demographics (sex and age group), details of the crime (type of crime, number of offenders, offender sex, offender relationship to the victim, and weapons used by the offender), and victim outcomes (reporting to police, injuries, lost work time, and lost pay because of lost workdays). Because detailed occupational information was only coded for victims reporting a workplace crime, rates of violent workplace crimes per 1,000 workers with 95% CIs were calculated for each of the 22 occupational groups using denominator occupation estimates from the U.S. Census Bureau’s Current Population Survey.^¶^ Occupation coding was only consistent between NCVS and the Current Population Survey during 2007–2010 for currently available data; therefore, rates were calculated only for this period. Estimates with small sample sizes were presented and flagged for reliability.** Analyses were conducted using SAS statistical software (version 9.4; SAS Institute).

During 2007–2015, an estimated 10.3 million violent crimes reported by persons aged ≥16 years occurred in the workplace, accounting for 22% of all violent crimes (95% CI = 20%–25%). During this period, approximately eight violent workplace crimes per 1,000 workers (95% CI = 7–9) were reported. During 2007–2010, occupations with the highest rates of violent workplace crimes were Protective services (e.g., first responders) (101 crimes per 1,000 workers); Community and social services (19); Healthcare practitioners and technicians (17), Healthcare support occupations (17); Education, training, and library occupations (eight); and Transportation and material moving occupations (seven) (Table 1).

**TABLE 1:** Nonfatal violent workplace crimes among persons aged ≥16 years, by occupation* — National Crime Victimization Survey (NCVS), United States, 2007–2010^†^

Occupation	Unweighted no.	Weighted no.	Rate^§,¶^ (95% CI)
Management	83	332,711	5.4 (3.7–7.8)
Business and financial operations	20	109,873**	4.5 (2.2–9.4)**
Computer and mathematical	<5	12,353**	0.9 (0.2–3.5)**
Architecture and engineering	<5	3,346**	0.3 (0.0–2.1)**
Life, physical, and social science	5	15,674**	2.9 (1.0–8.4)**
Community and social services	27	176,749**	19.1 (10.2–35.8)**
Legal	<5	2,999**	0.4 (0.1–3.1)**
Education, training, and library	82	262,633	7.6 (4.7–12.4)
Arts, design, entertainment, sports, and media	6	44,377**	4.0 (1.0–16.3)**
Healthcare practitioners and technical	74	515,456	17.1 (11.0–26.4)
Healthcare support	21	214,557**	16.5 (4.7–58.0)**
Protective services	136	1,274,811	101.4 (68.1– 151.0)
Food preparation and serving related	35	179,684**	5.8 (2.8–12.0)**
Building and grounds cleaning and maintenance	19	94,682**	4.4 (1.9–10.2)**
Personal care and services	11	54,028**	2.7 (1.1–6.8)**
Sales and related	87	338,450	5.3 (3.8–7.3)
Office and administrative support	48	267,987**	3.6 (1.9–6.8)**
Farming, fishing, and forestry	<5	5,532**	1.4 (0.3–5.9)**
Construction and extraction	21	79,159	2.4 (1.4–4.1)
Installation, maintenance, and repair	15	80,414**	4.0 (2.0–7.8)**
Production	23	79,201	2.3 (1.4–3.9)
Transportation and material moving	44	241,703	7.1 (4.1–12.2)

**Abbreviation:** CI = confidence interval.

* Free-text survey responses on occupation at the time of the workplace crime were categorized by NCVS into 44 nonmilitary occupational groups. These occupations were collapsed into 22 major groups defined by the U.S. Census Bureau.

^†^ Estimates by occupation could only be calculated for years 2007–2010 because this was the only period during which NCVS occupational coding was consistent with the U.S. Census Bureau’s Current Population Survey coding in the available data.

^§^ Crimes per 1,000 workers.

^¶^ Denominator estimate source is the U.S. Census Bureau’s Current Population Survey.

** Estimates were flagged for reliability if the unweighted frequency was <10 or the weighted frequency’s relative standard error was >30% of the weighted frequency, as recommended in a Bureau of Justice Statistics’ report, Evaluation of Direct Variance Estimation, Estimate Reliability, and Confidence Intervals for the National Crime Victimization Survey (https://www.bjs.gov/content/pub/pdf/edveercincvs.pdf). The observed values might have occurred because of chance or be unrepresentative of the general population.

Most workplace crimes were reported by men (63%) and persons aged 25–34 years (32%) (Table 2). The most frequently reported type of crime was threat of assault (44%), followed by simple assault (37%), aggravated assault (13%), rape/sexual assault (3%), and robbery (3%). Most violent workplace crimes involved male offenders, and approximately one in seven crimes involved a weapon. Fifty-eight percent of crimes were not reported to police. Fourteen percent of violent workplace crimes led to injury.

**TABLE 2:** Victim and crime characteristics of nonfatal violent workplace crimes, among persons aged ≥16 years overall and by victim sex — National Crime Victimization Survey, United States, 2007–2015

Characteristic	Overall			Male victims			Female victims		
	Unweighted no.	Weighted no.	Weighted %* (95% CI)	Unweighted no.	Weighted no.	Weighted %* (95% CI)	Unweighted no.	Weighted no.	Weighted %* (95% CI)
**Victim sex**									
Men	1,141	6,501,414	62.8 (58.4–67.0)	—	—	—	—	—	—
Women	807	3,844,447	37.2 (33.0–41.6)	—	—	—	—	—	—
**Age group (yrs**)									
16–24	223	1,417,731	13.7 (10.8–17.2)	131	752,834	11.6 (8.4–15.7)	92	664,898	17.3 (12.2–23.9)
25–34	519	3,302,795	31.9 (27.4–36.8)	340	2,358,872	36.3 (30.5–42.5)	179	943,924	24.6 (18.7–31.5)
35–44	484	2,565,956	24.8 (21.4–28.6)	306	1,784,028	27.4 (22.8–32.6)	178	781,928	20.3 (15.3–26.5)
45–54	432	1,895,648	18.3 (15.4–21.6)	220	991,694	15.3 (11.9–19.4)	212	903,954	23.5 (18.5–29.4)
≥55	290	1,163,731	11.2 (9.2–13.7)	144	613,987	9.4 (6.8–12.9)	146	549,744	14.3 (11.0–18.4)
**Type of crime**									
Threat of assault	828	4,542,664	43.9 (39.5–48.4)	514	3,166,482	48.7 (42.8–54.6)	314	1,376,182	35.8 (30.2–41.8)
Simple assault	706	3,799,570	36.7 (32.7–41.0)	377	2,124,891	32.7 (27.7–38.0)	329	1,674,679	43.6 (37.7–49.7)
Aggravated assault	304	1,390,667	13.4 (11.2–16.0)	190	951,052	14.6 (11.7–18.1)	114	439,615	11.4 (8.7–14.9)
Rape/Sexual assault	51	350,585	3.4 (2.0–5.8)	16	86,953^§^	1.3^§^ (0.5–3.3)	35	263,632^§^	6.9^§^ (3.6–12.7)
Robbery	59	262,375	2.5 (1.7–3.9)	44	172,036	2.6 (1.8–4.0)	15	90,339^§^	2.3^§^ (0.9–5.9)
**No. of offenders**									
Single	1,681	8,305,957	80.3 (76.5–83.6)	972	5,045,267	77.6 (73.1–81.5)	709	3,260,690	84.8 (79.4–89.0)
Multiple	171	1,321,050	12.8 (9.9–16.3)	101	877,175	13.5 (10.1–17.8)	70	443,875	11.5 (8.0–16.5)
Don't know	13	145,942^§^	1.4^§^ (0.6–3.1)	12	142,346^§^	2.2^§^ (1.0–4.9)	<5	3,596^§^	0.1^§^ (0.0–0.7)
**Offender’s sex**									
Male	1,431	7,323,470	70.8 (66.8–74.4)	940	5,008,418	77.0 (71.4–81.8)	491	2,315,052	60.2 (54.3–65.8)
Female	300	1,432,390	13.8 (11.4–16.7)	79	433,698	6.7 (4.3–10.3)	221	998,691	26.0 (21.8–30.7)
Male and female	45	372,505	3.6 (2.2–5.8)	18	156,792^§^	2.4^§^ (1.0–5.6)	27	215,714^§^	5.6^§^ (3.2–9.7)
Don't know	24	339,009^§^	3.3^§^ (1.8–6.0)	17	265,722^§^	4.1^§^ (2.0–8.2)	7	73,287^§^	1.9^§^ (0.6–5.5)
**Offender relationships** ^†^									
Work related	511	2,534,104	24.5 (20.8–28.6)	272	1,219,973	18.8 (15.0–23.2)	239	1,314,131	34.2 (27.0–42.2)
Customer/Client/Patient	180	1,178,467	11.4 (8.2–15.5)	79	443,352	6.8 (4.5–10.2)	101	735,116	19.1 (12.7–27.8)
Coworker	254	1,014,531	9.8 (7.8–12.2)	153	646,793	9.9 (7.3–13.5)	101	367,738	9.6 (7.1–12.8)
Supervisor	29	189,779^§^	1.8^§^ (0.9–3.9)	5	16,279^§^	0.3^§^ (0.1–0.7)	24	173,499^§^	4.5^§^ (2.0–9.9)
Employee	48	151,326	1.5 (1.0–2.0)	35	113,549	1.7 (1.2–2.6)	13	37,778	1.0 (0.5–1.8)
Relative or (ex) spouse/partner	32	146,452^§^	1.4^§^ (0.7–2.9)	7	21,492^§^	0.3^§^ (0.2–0.7)	25	124,960^§^	3.3^§^ (1.4–7.4)
Other known relationship	268	1,333,961	12.9 (10.4–15.9)	110	631,760	9.7 (6.9–13.4)	158	702,200	18.3 (13.8–23.8)
Recognized but unknown	254	1,450,801	14.0 (11.1–17.6)	146	961,622	14.8 (10.6–20.3)	108	489,180	12.7 (9.1–17.4)
Stranger	658	3,760,919	36.4 (32.5–40.4)	460	2,826,593	43.5 (38.5–48.6)	198	934,326	24.3 (19.6–29.8)
**Weapons**									
No weapon	1,518	8,361,178	80.8 (78.0–83.4)	861	5,107,562	78.6 (74.7–82.0)	657	3,253,616	84.6 (80.8–87.8)
Weapon^†^	324	1,489,692	14.4 (12.0–17.2)	208	1,041,583	16.0 (12.8–19.9)	116	448,109	11.7 (8.8–15.2)
Firearm	101	404,761	3.9 (2.8–5.5)	70	311,645	4.8 (3.1–7.3)	31	93,117	2.4 (1.6–3.7)
Knife/Sharp object	92	394,670	3.8 (2.8–5.2)	65	298,854	4.6 (3.2–6.5)	27	95,816^§^	2.5^§^ (1.3–4.9)
Blunt object	61	337,323	3.3 (2.3–4.7)	36	225,116	3.5 (2.1–5.6)	25	112,207^§^	2.9^§^ (1.5–5.5)
Other	80	436,242	4.2 (2.9–6.1)	44	281,499	4.3 (2.5–7.3)	36	154,743	4.0 (2.6–6.3)
Don't know	106	494,991	4.8 (3.4–6.7)	72	352,268	5.4 (3.5–8.3)	34	142,722	3.7 (2.2–6.2)
**Crime reported to police**									
No	1,057	5,961,317	57.6 (53.4–61.8)	579	3,521,257	54.2 (48.3–59.9)	478	2,440,061	63.5 (57.3–69.2)
Yes	818	4,021,869	38.9 (34.8–43.1)	517	2,701,320	41.5 (35.9–47.4)	301	1,320,549	34.3 (28.6–40.6)
Don't know	44	273,376	2.6 (1.5–4.6)	33	236,237^§^	3.6^§^ (1.9–6.7)	11	37,139^§^	1.0^§^ (0.5–1.8)
**Injuries**									
No	1,689	8,947,068	86.5 (82.9–89.4)	1,006	5,738,080	88.3 (83.6–91.7)	683	3,208,989	83.5 (78.1–87.7)
Yes	259	1,398,793	13.5 (10.6–17.1)	135	763,335	11.7 (8.3–16.4)	124	635,458	16.5 (12.3–21.9)
**Any work time lost due to incident**									
No	1,758	9,358,340	90.5 (87.8–92.6)	1,045	6,036,906	92.9 (90.2–94.8)	713	3,321,434	86.4 (80.2–90.9)
Yes^†^	190	987,521	9.5 (7.4–12.2)	96	464,508	7.1 (5.2–9.8)	94	523,014	13.6 (9.1–19.8)
Due to injuries	65	289,877	2.8 (1.8–4.3)	30	153,376	2.4 (1.3–4.1)	35	136,501^§^	3.6^§^ (1.9–6.5)
Due to police or court activities	63	431,471	4.2 (2.5–6.8)	38	215,965	3.3 (1.9–5.7)	25	215,506^§^	5.6^§^ (2.3–12.9)
Due to other reasons	78	347,737	3.4 (2.4–4.7)	37	123,828	1.9 (1.3–2.8)	41	223,909	5.8 (3.6–9.4)
**Pay lost from lost workdays** ^¶^									
No lost pay for missed days	82	502,274	4.9 (3.1–7.4)	41	223,433	3.4 (2.1–5.5)	41	278,841^§^	7.3^§^ (3.6–13.9)
Lost pay for missed days	59	274,864	2.7 (1.7–4.1)	22	83,222	1.3 (0.8–2.1)	37	191,642	5.0 (2.8–8.8)

**Abbreviation:** CI = confidence interval.

* Percentages represent the proportion of all nonfatal violent workplace crimes; percentages might not sum to 100% because of missing values or non–mutually exclusive groups.

^†^ Incidents are not mutually exclusive and might fall into more than one category.

^§^ Estimates were flagged for reliability if the unweighted frequency was <10 or the weighted frequency’s relative standard error was >30% of the weighted frequency, as recommended in a Bureau of Justice Statistics’ report, Evaluation of Direct Variance Estimation, Estimate Reliability, and Confidence Intervals for the National Crime Victimization Survey (https://www.bjs.gov/content/pub/pdf/edveercincvs.pdf). The observed values might have occurred because of random chance or be unrepresentative of the general population.

^¶^ A total of 1,807 respondents who lost time following the crime did not lose at least 1 full day.

When stratified by victim sex, the most prevalent type of crime against men was threat of assault (49%) and against women, was simple assault (44%). Women reported higher proportions of crimes committed by offenders known from the workplace than did men (34% versus 19%), including customers/clients/patients (19% [women] versus 7% [men]); 43% of violent workplace crimes reported by men were committed by strangers, compared with 24% reported by women. The proportion of violent workplace crimes leading to lost pay because of lost workdays was higher among women than among men (5% versus 1%).

## Discussion

Violent workplace crimes were reported by U.S. workers in all occupational groups during 2007–2010. During 2007–2015, approximately eight nonfatal workplace crimes per 1,000 workers were reported, and 58% of crimes were not reported to police. Highest rates of crime were among Protective services, Community and social services, and Healthcare occupations. More crimes against women than men were reportedly committed by offenders known from the workplace. Findings demonstrated that the prevalence, characteristics, and outcomes of violent workplace crime varied by occupation and victim sex.

A recent NCVS analysis estimated rates of violent workplace crimes by selected occupations only and reported an overall crime rate of approximately four crimes per 1,000 workers in 2009 (*7*). However, the analysis did not include threats of assault, which are categorized by the Occupational Safety and Health Administration (OSHA) as workplace violence and can also lead to adverse physical and psychological health outcomes (*1*,*8*). Other national estimates of nonfatal workplace violence often rely on workers’ compensation claims, emergency department data, or employer-reported injuries leading to lost work time, which underestimate the actual prevalence of workplace violence (*2*–*4*). Self-reported responses provide information on crimes that might not have been reported to employers or police or that do not lead to injury; most violent workplace crimes in NCVS were not reported to police.

The highest rates of nonfatal workplace violence were found among Protective services; Community and social services; Healthcare; Education; and Transportation occupational groups. These findings are consistent with other studies finding high rates of workplace violence in these groups (*2*,*4*,*7*,*9*). High-risk occupations appear to be those most likely to involve interpersonal contact, especially with persons who might be violent, upset, or vulnerable. This analysis identified some differences between male and female victims of nonfatal workplace violence that have not been evaluated in recent years, including the type of crime, relationship to the offender, and impact on pay. Although few studies have examined sex differences in characteristics of violent workplace crimes, some suggest that inequalities can be partly attributed to sex differences in work hours/shifts, conflict-resolution strategies, and work assignments based on social roles (*3*,*10*).

The findings in this report are subject to at least three limitations. First, only 1,948 violent workplace crimes (unweighted) were reported in NCVS for the years 2007–2015. Small sample sizes yielded many estimates that were flagged for reliability. Second, self-reported crime information can be inaccurate. Stigma or safety issues (e.g., intimate partner violence if the offender was in the household) might have discouraged or prevented persons from accurately reporting victimization. Misclassification might have occurred among offender relationship types if victims reported offenders that were patients/clients/customers as strangers. Finally, the period of recall for crimes was 6 months, which might have led to inaccurate recollection if crimes occurred months before survey administration or the incident was perceived to be relatively minor.

These findings demonstrate that the incidence of nonfatal workplace violence is likely an underreported public health issue that varies by worker and work characteristics. Workplace violence prevention programs might benefit from having different approaches or components for specific worker groups based on different offender relationships. A previous NCVS supplement on workplace violence revealed that only 60% of respondents reported that their employer had written guidelines regarding workplace violence. Fewer than one third of respondents had ever participated in a workplace violence prevention training (*9*). Workplace violence falls under OSHA’s General Duty Clause that states all workers have the right to a safe work environment. OSHA recommends engineering controls, administrative controls, employee training, and zero-tolerance policies toward workplace violence (*8*). Training and controls should emphasize how employers and employees can recognize and manage specific risk factors in prevention programs. Future research could investigate underlying reasons for sex differences in workplace violence and effective methods for preventing and managing workplace violence hazards.

**Summary**

**What is already known about this topic?**

Workplace violence can lead to adverse health and work outcomes. Few data sources are available for estimating national prevalence of nonfatal workplace violence.

**What is added by this report?**

Approximately eight nonfatal violent workplace crimes were reported per 1,000 U.S. workers during 2007–2015; 58% of crimes were not reported to police. Highest rates of crime were among Protective services, Community and social services, and Healthcare occupations. More crimes against women than men were reportedly committed by offenders known from the workplace.

**What are the implications for public health practice?**

The incidence of nonfatal workplace violence varies by worker characteristics. Violence prevention programs might benefit from having different approaches for specific worker groups.

## Footnotes

* Data used are from Version 1, which has since been revised as Version 3.

† Types of crime included rape/sexual assault (including attempted rape, sexual attack with serious/minor assault, sexual assault without injury, unwanted sexual contact without force, and verbal threat of rape/sexual assault); robbery (including attempted robbery); aggravated assault (attack or attempted attack with a weapon, regardless of whether or not an injury occurred, and attack without a weapon when serious injury resulted, and including aggravated assault with injury, attempted aggravated assault with weapon, and threatened assault with weapon); simple assault (attack without a weapon resulting in no or minor injury, and including simple assault with injury and assault without weapon without injury); and verbal threat of assault.

§ The NCVS classified nonmilitary occupations as one of 22 major groups or 20 minor groups of special interest to the Bureau of Justice Statistics as defined by the U.S. Census Bureau. Analyses further classified the NCVS groups as follows: Management; Business and financial operations; Computer and mathematical; Architecture and engineering; Life, physical, and social science; Community and social services (including social workers); Legal; Education, training, and library (including preschool (prekindergarten and kindergarten), elementary, junior high or middle school, high school, college or university, technical or industrial school, special education facilities); Arts, design, entertainment, sports, and media; Healthcare practitioners and technical (including physicians, nurses, health technicians); Healthcare support (including healthcare aides); Protective services (including police officers, prison or jail guards, security guards); Food preparation and serving related; Building and grounds cleaning and maintenance; Personal care and services; Sales and related (including grocery, convenience, or liquor store clerks, gas station attendants, bartenders); Office and administrative support; Farming, fishing, and forestry; Construction and extraction; Installation, maintenance, and repair; Production; Transportation and material moving (including bus drivers, taxi cab drivers and chauffeurs).

¶ U.S. Census Bureau’s Current Population Survey occupation estimates were obtained from the CDC’s Employed Labor Force (ELF) query system (https://wwwn.cdc.gov/wisards/cps/cps_estimates.aspx). Estimates for 2007–2010 were coded in the ELF system according to 2002 U.S. Census Bureau occupational classification.

** Estimates were flagged for reliability if the unweighted frequency was <10 or the weighted frequency’s relative standard error was >30% of the weighted frequency, as recommended in a Bureau of Justice Statistics’ report, Evaluation of Direct Variance Estimation, Estimate Reliability, and Confidence Intervals for the National Crime Victimization Survey (https://www.bjs.gov/content/pub/pdf/edveercincvs.pdf). The observed values might have occurred because of chance or be unrepresentative of the general population.
